# Soy Protein-Based Emulsions: Application as Lipid Substitutes in Surimi Gels

**DOI:** 10.3390/foods14132342

**Published:** 2025-07-01

**Authors:** Fali Zhang, Jian Shi, Yanfei Chen, Yao Yue, Wenzheng Shi, Tanye Xu, Min Qu

**Affiliations:** 1 College of Food Science and Engineering, Dalian Ocean University, Dalian 116023, China; farleigh.z@foxmail.com (F.Z.); xutanye@dlou.edu.cn (T.X.); 2 College of Food Science and Technology, Shanghai Ocean University, Shanghai 201306, China; s19895905220@163.com (J.S.); wzshi@shou.edu.cn (W.S.); 3 College of Fisheries and Life Sciences, Dalian Ocean University, Dalian 116023, China; 15941016593@163.com; 4 School of Pharmacy, East China Normal University, Shanghai 200062, China; yauyiu@foxmail.com; 5 School of Computer Science and Technology, East China Normal University, Shanghai 200062, China

**Keywords:** surimi products, lipid substitutes, Pickering emulsion, soy protein, rheological properties

## Abstract

By analyzing interfacial dynamics between soybean oil concentrations and soy protein isolate (SPI), this study established their impact on Pickering emulsion stability. Two optimal soy protein-based emulsions (EM60 with 60% oil phase; EM75 with 75%) were identified as lipid substitutes in silver carp surimi products. The results revealed that uniformly spherical droplets in EM60 enhanced interparticle interactions at emulsion interfaces. Compared to EM75 addition, EM60’s finely dispersed droplets improved gel network compactness in the surimi matrix. This increased water-holding capacity (WHC) by 12.037% and gel strength by 2414.168 g·mm. EM75 addition significantly enhanced gel whiteness by 0.8483 units (*p* < 0.05). It also demonstrated superior physical filling effects in sol state, reinforcing structural rigidity. As unsaturated lipids, soybean oil substitution for saturated fats (e.g., lard) contributes positively to human health. Pre-emulsified soybean oil yielded stronger structural rigidity in surimi sol than direct oil addition. Post-gelation, significant increases were observed in gel strength (+828.100 g·mm), WHC (+6.093%), and elasticity (+0.07 units). Collectively, SPI-based emulsions offer novel insights for healthy lipid substitution in surimi gels. They elucidate differential impact mechanisms on texture, WHC, whiteness, and microstructure. This provides theoretical guidance for developing premium healthy surimi products.

## 1. Introduction

The silver carp (*Hypophthalmichthys molitrix*), native to Southeast Asia, ranks among the most extensively farmed freshwater fish species worldwide. Together with bighead carp (*Hypophthalmichthys nobilis*), grass carp (*Ctenopharyngodon idella*), and snakehead (*Channa argus*), they constitute the “Four Major Chinese Cyprinids”. Characterized by low production costs and high farming efficiency, silver carp has become one of the most globally prevalent aquaculture species [1]. With high white muscle yield, low endogenous cathepsin activity, and rich nutritional profile, silver carp serves as an excellent raw material for surimi production [2].

Surimi products, traditional East Asian delicacies, are processed through deboning, washing, and comminution to create high-protein, low-fat aquatic foods [3]. Myofibrillar proteins, primarily myosin and actin complexes, play pivotal roles in surimi gelation. Thermal denaturation induces irreversible aggregation of these proteins, forming crosslinked three-dimensional networks through disulfide bonding. Consequently, gel properties serve as critical quality indicators for surimi products.

While washing enhances gel characteristics and storage stability during surimi processing, it simultaneously removes lipid components responsible for product-specific flavor profiles [4]. Although lipids contribute desirable textural attributes like smoothness and tenderness, excessive unsaturated fat intake raises health concerns amidst growing nutritional awareness. Current practices employ plant oils (peanut, corn, soybean, and camellia oil) as exogenous additives in surimi products to substitute saturated fats, aiming to improve color, gel properties, and flavor characteristics. However, studies demonstrate that direct oil incorporation disrupts myofibrillar protein interactions, yielding heterogeneous gel networks with coarse, porous microstructures that compromise product quality [5,6]. Pre-emulsification of natural oils with surface-active protein particles to form Pickering emulsions prior to gel incorporation significantly mitigates these textural deteriorations. 

Emulsion classification depends on oil phase content: systems with oil volume fractions > 74% (*v*/*v*) constitute high internal phase emulsions (HIPEs), while those ≤74% represent conventional Pickering emulsions [7] HIPEs exhibit superior lipid-loading capacities. Sun et al. [8] developed casein-stabilized HIPEs with Litsea cubeba oil, demonstrating significant improvements in silver carp surimi gel texture and flavor profiles. Zhang et al. [9] revealed that soybean oil converted into HIPEs effectively counteracts the detrimental effects of free oil droplets on surimi gel properties.

Current research predominantly focuses on the effects of pre-emulsified natural oils as high internal phase emulsions (HIPEs) on surimi gel properties, while comparative studies between low internal phase emulsions (LIPEs) and HIPEs in surimi gel systems remain scarce. In particular, investigations into emulsions with varying oil phase contents as lipid substitutes in surimi gels are still limited. Furthermore, soy protein particles containing both 11S and 7S globulins have been demonstrated to exhibit enhanced interfacial activity, enabling the formation of stabilized interfacial layers at oil–water interfaces [10]. Therefore, this study aimed to evaluate the effects of soy protein-stabilized Pickering emulsions with different oil-phase ratios (60% and 75%) on the physical, rheological, and microstructural properties of silver carp surimi gels. Comparative analyses were conducted against control groups with conventional lipid addition to assess potential as healthier fat substitutes.

## 2. Materials and Methods

### 2.1. Materials

Frozen silver carp (*Hypophthalmichthys molitrix*) surimi (AAA grade, containing 74.97% *w*/*w* moisture and 14.41% *w*/*w* protein) was purchased from Honghu Aquatic Products Co., Ltd. (Honghu Aquatic Products Co., Ltd., Honghu, China). Soybean oil was obtained from Yihai Kerry Oils & Grains Industries Co., Ltd. (Wuhan, China). Analytical-grade soy protein, sodium chloride, and other chemicals were sourced from Yuanye Bio-Technology Co., Ltd. (Yuanye Bio-Technology Co., Ltd., Shanghai, China).

### 2.2. Emulsion Preparation

Soy protein samples were blended with deionized water to achieve a final concentration of 10% (*w*/*w*). Soybean oil was dispersed in soy protein isolate suspensions at varying oil volume fractions (10%, 20%, 40%, 60%, 75%). The mixtures were homogenized using a T25 easy clean digital Ultra-Turrax (IKA Co., Ltd., Staufen, Germany) at 10,000 rpm for 2 min, producing soy protein-stabilized emulsions designated as EM10, EM20, EM40, EM60, and EM75 based on oil content. All emulsions were stored at 4 °C for subsequent use.

Table 1 shows the summary of protein and oil contents in the emulsions.

### 2.3. Emulsion Characterization

#### 2.3.1. Macrostability and Creaming Index Analysis

Emulsions were transferred to glass vials and stored at 4 °C for 35 days. Macroscopic morphology and serum/cream layer heights were recorded at 25 °C on days 1, 3, 7, 14, and 35 during storage. The creaming index (CI) was calculated using Equation (1) to evaluate storage stability [11]:

(1) $$CI=\frac{HS}{HE}\times 100$$

where *H_S_* and *H_E_* represent cream layer height and total emulsion height (mm), respectively, measured with digital calipers.

#### 2.3.2. Optical Microscopy

Microstructural analysis was performed following R. Afkhami et al. [12] using optical microscopy (Minz Precision Instruments Co., Ltd., Shanghai, China) with 10× objective at specified intervals (1, 3, 7, 14, and 35 days).

#### 2.3.3. Confocal Laser Scanning Microscopy (CLSM)

Emulsions were dual-stained with Nile Red and Nile Blue, then equilibrated for 30 min. Oil–water distribution was analyzed using a Leica TCS SP8 CLSM (Leica Microsystems Trading Co., Ltd., Wetzlar, Germany) with 20× objective, employing excitation wavelengths of 488 nm and 633 nm.

#### 2.3.4. Rheological Characterization of Emulsions

##### Steady Shear Viscosity

Steady shear measurements were conducted using a rotational rheometer (Discovery DHR-2, TA Co., Ltd., Newcastle, DE, USA) equipped with 40 mm parallel plates, with shear rates ranging from 0.1 to 100 s^−1^.

##### Strain Amplitude Sweep

Strain sweeps (0.1–100% strain) were performed at 1 Hz frequency and 25 °C to determine storage modulus (G′) and loss modulus (G″).

##### Frequency Sweep

Frequency-dependent viscoelastic properties were analyzed through oscillatory tests (0.1–100 rad/s) at 0.1% strain and 25 °C.

##### Alternating Strain Test

Eight alternating strain cycles (0.1% ↔ 100%, 120 s/cycle) were applied at 1 Hz and 25 °C to monitor G′ and G″ responses under varying deformation conditions.

### 2.4. Surimi Gel Preparation

Frozen surimi was thawed at 4 °C overnight, then mixed with 2.5% NaCl (*w*/*w*) and ground for 1 min. Pre-prepared emulsions containing 60% and 75% oil phase (EM60 and EM75) were incorporated at 5%, 10%, and 15% (*w*/*w*) levels. The mixtures were comminuted for 2 min at 4 °C using a silent cutter, maintaining final moisture content at 78% (*w*/*w*). Resulting samples were designated as E60-5, E60-10, E60-15, E75-5, E75-10, and E75-15 based on emulsion type and incorporation level. Control groups included E60-10 counterpart with 6% soybean oil + 4% soy protein, and E0 (blank control) without emulsion or oil addition [13]. The samples were ultimately categorized into two groups: One group comprised the gel state obtained post-thermal processing. The other group constituted the sol state, both designated for subsequent characterization.

### 2.5. Surimi Gel Quality Evaluation

#### 2.5.1. Color Analysis

Color parameters (L*, a*, b*, chroma, whiteness) were measured using a CR-10 colorimeter (Konica Minolta Investment Co., Ltd., Tokyo, Japan), where L* indicates lightness, a* red-green axis, and b* yellow-blue axis. Gels were sectioned into 5 mm slices and measured six times on surface areas at room temperature. Following white plate calibration, whiteness (W) was calculated using Equation (2) [14]:

(2) $$W=100-\sqrt{{\left(100-L^{*}\right)}^{2}+{a^{*}}^{2}+{b^{*}}^{2}}$$

#### 2.5.2. Texture Profile Analysis (TPA)

Surimi gels were equilibrated to 25 °C and sectioned into 20 mm height cylinders (TA-XT. plus, Stable Microsystem Co., Ltd., Surry, UK) for texture analysis using a texture analyzer. A P/50 cylindrical probe performed two-cycle compression tests to determine hardness (g), springiness, cohesiveness, and chewiness (g). Testing parameters included pre-test speed 3.00 mm/s, test speed 1.00 mm/s, post-test speed 5.00 mm/s, 40% deformation, and 5 g trigger force.

#### 2.5.3. Gel Strength Evaluation

Gels were temperature-equilibrated at room temperature for 30 min and shaped into 25 mm-height cylinders. A TA-XT Plus texture analyzer with P/5S spherical probe measured fracture force (g) and deformation distance (mm) for gel strength calculation. esting parameters: 1.00 mm/s speed, 15 mm penetration depth, 10 g trigger force. Gel strength was calculated using Equation (3):

*Gel strength* (g·mm) = *Fracture force* (g) × *Deformation distance* (mm) (3)

#### 2.5.4. Water-Holding Capacity (WHC)

Following Fan et al. [15], gel samples (5 mm cubes, initial weight m_1_) were centrifuged at 10,000 rpm for 15 min in filter paper. Post-centrifugation weight (m_2_) was recorded, with WHC calculated using Equation (4):

(4) $$WHC\left(\%\right)=\frac{m_{2}}{m_{1}}\times 100$$

#### 2.5.5. Low-Field Nuclear Magnetic Resonance (LF-NMR)

T_2_ relaxation times were measured using a MesoMR23-060H-I LF-NMR analyzer (Niumag Co., Ltd., Suzhou, China) following Zhang et al. [16]. Uniform cylindrical samples (20 mm height × 25 mm diameter) were positioned on the MRI platform. Eight scans at 32 °C captured 8000 echoes with 21 MHz resonance frequency. T_2_ spectra and peak area ratios (P_2_) were obtained via MultiExpInv software inversion.

#### 2.5.6. Magnetic Resonance Imaging (MRI)

MRI analysis was adapted from Traffano-Schiffo et al. [17] with modifications. Cylindrical samples (20 mm height × 25 mm diameter) were loaded into MRI tubes. Acquisition parameters: TE 18.2 ms, TR 500 ms. Proton density maps were generated using MRI software.

#### 2.5.7. Rheological Properties of Surimi Sol

Rheological properties of surimi sol were analyzed using a rotational rheometer (Discovery DHR-2, TA Co., Ltd., Newcastle, DE, USA) equipped with 40 mm parallel plates, following the method of Liu et al. [18].

##### Apparent Shear Viscosity

Apparent shear viscosity was measured at shear rates ranging from 0.1 to 100 s^−1^ at 25 °C. The data were fitted to the Ostwald–de Waele model (Equation (5)),

(5) $$\eta =k{\left(\gamma \right)}^{n-1}$$

where *η* (Pa·s) is the apparent shear viscosity, *γ* (s^−1^) is the shear rate, *k* (Pa·s^n^) is the consistency coefficient, and (n) is the flow behavior index.

##### Oscillatory Amplitude Strain and Frequency Sweep

At a fixed frequency of 1 Hz and 25 °C, storage modulus (G′) and loss modulus (G″) were measured over a strain range of 1% to 1000% to determine the linear viscoelastic region (LVR).

Frequency sweeps were conducted within the LVR (1%) to measure G′ and G″ at angular frequencies ranging from 1 to 100 rad/s. The results were fitted to the power-law models (6) and (7).

(6) $$G^{\prime }=a{\left(\omega \right)}^{b}$$

where (*a*) and (*b*) are constants dependent on the dynamic rheological behavior of the sample. For purely elastic gels, (*b* = 0), and for viscoelastic gels, (*b* > 0).

(7) $$G^{*}=t{\left(\omega \right)}^{\frac{1}{z}}$$

here, *G∗* refers to the complex shear modulus (*G∗* = *G′*^2^ + *G″*^2^)^1/2^, *ω* is the angular frequency, *t* reflects the gel strength, and *z* reflects the network extension of the structure [1].

##### Temperature Sweep

A temperature sweep was performed at a fixed frequency of 1 Hz and 1% strain, with a heating rate of 2 °C/min from 25 °C to 90 °C, to monitor the changes in G′ and G″ as the surimi sol transitioned to a gel state.

#### 2.5.8. Optical Microscopy of Surimi Gel

Following a slight modification of the method given by Jia et al. [19], the prepared surimi gel samples were sectioned into 5 × 5 × 2 mm rectangular blocks, fixed in glutaraldehyde solution, and washed once with phosphate-buffered saline. Dehydration and defatting were performed sequentially in ethanol solutions of 30%, 50%, 60%, 70%, 80%, 90%, and 100%, followed by tert-butanol. The samples were then cooled to −20 °C and sectioned to 4 μm thickness. Tissue sections were stained with 0.1% eosin and examined under an ML8000 microscope (Minz Precision Instruments Co., Ltd., Shanghai, China) equipped with a 10× objective lens), and images were recorded.

### 2.6. Statistical Analysis

All experiments were performed in triplicate independent trials. Data analyses were conducted using SPSS 22.0 (SPSS Statistics, IBM Corporation, Armonk, NY, USA). Significant differences between means were determined by Duncan’s multiple range test at the 5% significance level (*p* < 0.05).

## 3. Results and Discussion

### 3.1. Physical Stability of Pickering Emulsions

#### 3.1.1. Optical Microscopy and Creaming Index

**Figure 1:**
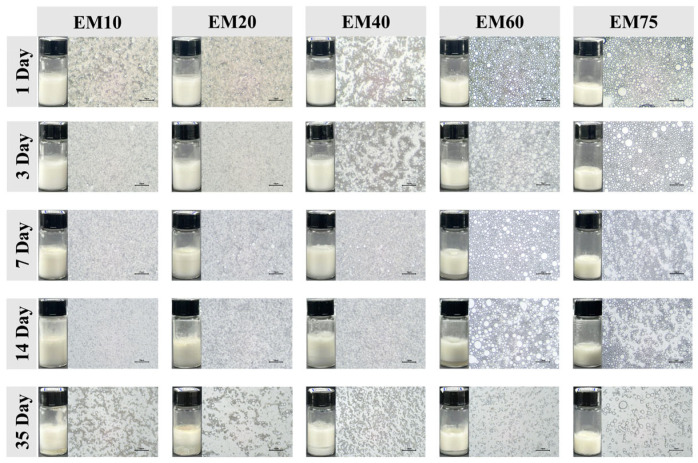
Macroscopic stability and microscopic structure of soy protein-stabilized emulsions under different oil phase contents.

Microscopic images of Pickering emulsions are commonly used to evaluate emulsion stability [20]. As shown in Figure 1, during 35 days of storage, as the oil phase content in the emulsion increased, the emulsion droplets became more rounded and uniformly dispersed, with smaller interdroplet spacing. However, as the storage time increased, emulsions with 10%, 20%, and 40% oil phase content at 35 days exhibited varying degrees of Ostwald ripening, coalescence, and flocculation. In contrast, the EM60 and EM75 emulsions maintained more intact droplet shapes and uniform dispersion, demonstrating the best resistance to Ostwald ripening [21].

**Figure 2:**
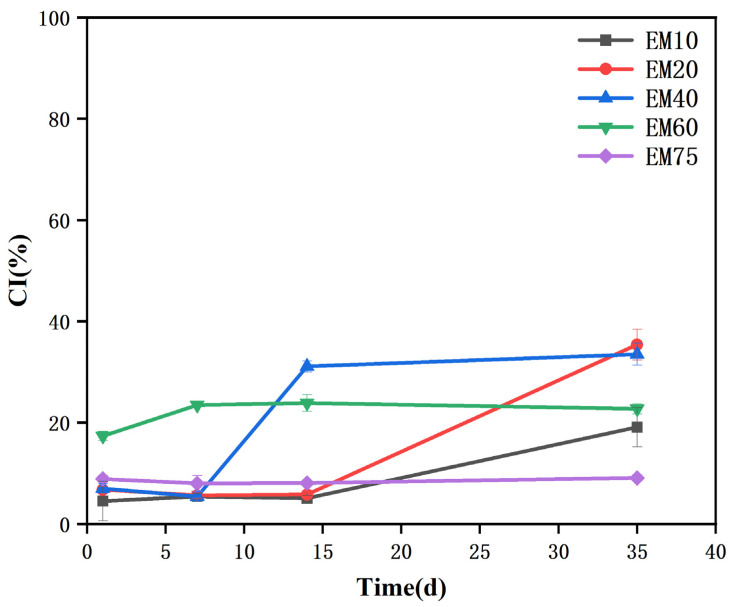
Creaming index of soy protein-stabilized emulsions with varying oil phase contents.

Figure 2 shows the changes in the creaming index (CI) of soy protein emulsions with different oil phase contents over time. All emulsions exhibited good stability within the first 7 days of storage. However, the EM40 emulsion started to show phase separation after 7 days, and the EM10 and EM20 emulsions followed suit after 14 days, with a significant increase in the creaming index (CI). The creaming index of the EM60 emulsion did not significantly increase after 7 days (*p* > 0.05), maintaining a stable trend. The EM75 emulsion did not exhibit any phase separation over the 35-day storage period (Figure 1), and its creaming index remained unchanged. Both figures indicate that the EM75 emulsion exhibited the best storage stability and emulsifying performance, followed by the EM60 emulsion. This is attributed to the appropriate oil phase content, which allows the oil droplets to maintain a uniform and complete shape, better filling the gel network of the emulsion and thus enhancing its stability.

#### 3.1.2. Confocal Laser Scanning Microscopy (CLSM)

**Figure 3:**
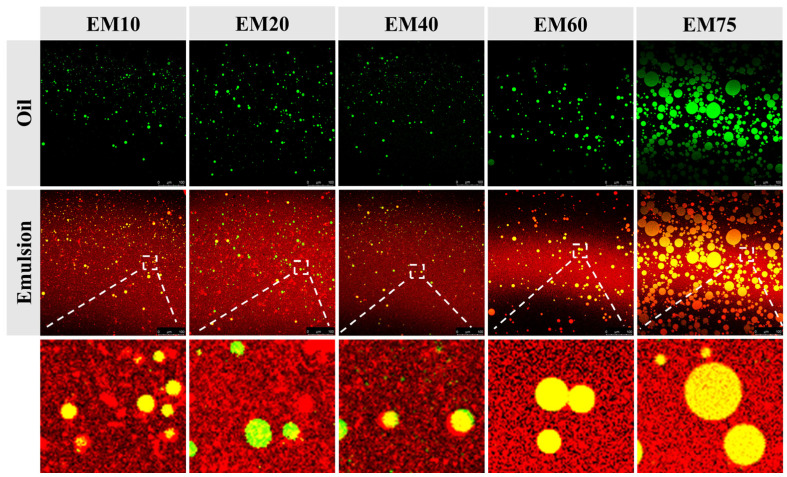
Water–oil phase distribution in soy protein-stabilized emulsions with different oil phase contents.

The proteins and oil phase in the emulsion were stained with Nile Red and Nile Blue, respectively (red indicates the presence of protein, green indicates the oil phase). The oil droplets in the emulsion appear green, surrounded by red soy protein particles. In Figure 3, all emulsion droplets did not exhibit Ostwald ripening or coalescence. This is attributed to the good hydrophilic and hydrophobic properties of the natural biopolymer particles, which allow the soy protein particles to form crosslinks between droplets and adsorb onto the droplet surfaces, thereby enhancing emulsion stability [22]. However, as the oil phase content in the emulsion increased, the oil droplets in EM60 and EM75 emulsions became more uniform and rounded. This is due to the increasing interactions between the protein particles and the droplet surfaces, leading to enhanced emulsion stability. This observation is consistent with the results from optical microscopy and creaming index measurements.

At oil phase contents of 10%, 20%, and 40%, there are more overlapping and adsorbed protein molecules at the emulsion interface. The resulting electrostatic repulsion may cause deformation and non-uniformity in the droplet shapes, leading to significant changes in the creaming index [23].

Additionally, previous studies have shown that in high internal phase emulsions, the reduction in protein particle coverage can lead to an increase in droplet size and a higher likelihood of droplet collisions, resulting in interdroplet compression and deformation [24,25]. Therefore, within a reasonable range of protein particle concentrations, an appropriate oil phase content allows the particles to adsorb at the droplet interfaces, forming a stable interfacial film, thus achieving the stabilization of Pickering emulsions.

### 3.2. Rheological Properties of Emulsions

**Figure 4:**
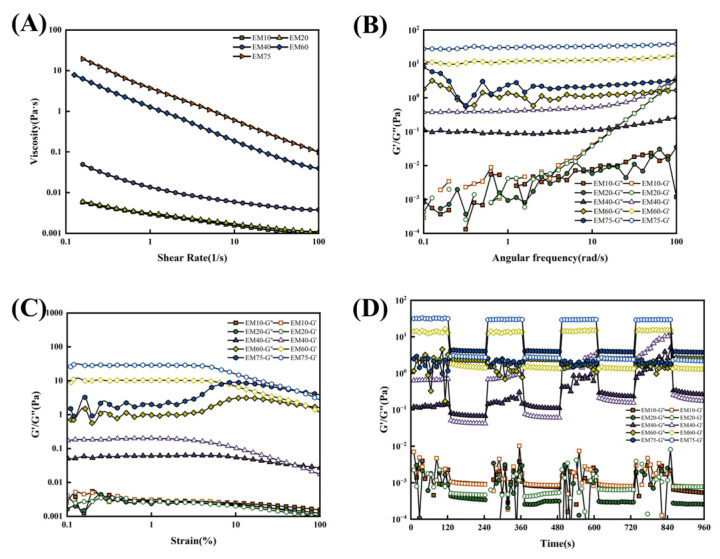
Rheological properties of soy protein-stabilized emulsions: (**A**) Shear viscosity sweep; (**B**) frequency sweep; (**C**) oscillatory strain sweep; (**D**) alternating strain sweep.

Storage modulus (G′) and loss modulus (G″) are critical parameters for evaluating material viscoelasticity. G′ represents energy storage during deformation, while G″ characterizes energy dissipation through viscous response. As shown in Figure 4A, increasing shear rates disrupted intermolecular interactions, leading to structural breakdown and progressive viscosity reduction [26]. This shear-thinning behavior confirms the soy protein emulsion as a classical non-Newtonian pseudoplastic fluid [27]. Notably, the viscosity exhibited consistent enhancement with increasing oil-phase ratios across all shear rates, with high internal phase emulsion (EM75) demonstrating optimal viscosity performance. These observations indicate that higher oil-phase ratios improve the viscoelastic properties of soy protein-based emulsions.

Figure 4C demonstrates proportional increases in both G′ and G″ with elevated oil phase content. Under low strain conditions, all emulsions displayed linear viscoelastic behavior, with yield points (G′-G″ crossover) sequentially emerging as strain increased. EM10 and EM20 showed comparable G′ and G″, while EM40, EM60, and EM75 maintained G′ > G″, indicating stronger gel networks and Pickering emulsion-like solid characteristics at 40%, 60%, and 75% oil-phase ratios [28]. Higher oil-phase emulsions (EM40/60/75) exhibited delayed yield point emergence and broader linear viscoelastic regions (LVR), particularly in EM60/75.

Frequency sweeps at 0.1% strain (Figure 4B) revealed minimal angular frequency dependence in EM60/75, suggesting stable particle adsorption at oil–water interfaces with superior self-recovery capacity. EM40 showed intermediate sensitivity, while EM10/20 exhibited significant G′-G″ fluctuations at low frequencies (indicating vulnerable intermolecular forces) and viscous dominance (G″ > G′) at high frequencies, reflecting inferior stability [29]. Alternating strain sweeps (Figure 4D) confirmed enhanced G′ and G″ with higher oil phase content, consistent with frequency/strain sweep observations. The emulsions behaved as solid-like (G′ > G″) at low strains and viscous fluids at high strains. EM60/75 maintained stable modulus relationships during prolonged testing, demonstrating excellent self-recovery properties [30].

### 3.3. Color Characteristics of Surimi Gels

Color serves as a critical visual sensory attribute of food, directly influencing consumer acceptance of products. The L*, a*, and b* values represent lightness, red-green chromaticity, and yellow-blue chromaticity, respectively [31]. As shown in the figure, both oil phase addition and emulsion-incorporated surimi gels exhibited significantly higher L* and a* values compared to the control group E0 (*p* < 0.05). The L* values of surimi gels increased progressively with emulsion concentration. While no significant difference was observed between 10% and 15% emulsion additions in the E75 group (*p* > 0.05), these values were significantly higher than those of the E60 group (*p* < 0.05). At 5% emulsion addition, no significant difference was detected between E75 and E60 groups. The E60-10 emulsion group showed significantly higher a* values than the direct oil phase addition group (E0-OIL) (*p* < 0.05), though no differences were observed in L* and b* values (*p* > 0.05).

**Figure 5:**
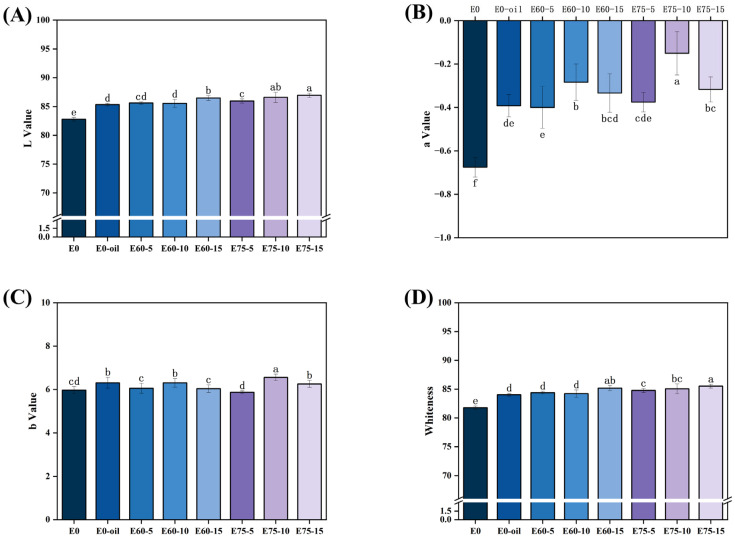
Chromaticity changes in surimi gels with different emulsion additives. Different lowercase letters within the same column indicate statistically significant differences (*p* < 0.05). (**A**) L* value (lightness); (**B**) a* value (red-green chromaticity); (**C**) b* value (yellow-blue chromaticity); (**D**) the whiteness index.

Whiteness serves as a critical sensory indicator for surimi product quality, with higher whiteness values correlating positively with consumer preference [32]. As demonstrated in Figure 5D, direct soybean oil addition and pre-emulsified oil delivery both significantly enhanced the whiteness of surimi gels (*p* < 0.05) owing to the light-scattering effect of dispersed droplets that masks chromophoric components. At 5% and 10% addition levels, the E75 group exhibited significantly higher whiteness than E60 (*p* < 0.05) whereas no significant difference emerged between E75 and E60 gels at the critical concentration (*p* > 0.05). Within the effective dosage range, whiteness demonstrated a concentration-dependent positive correlation with emulsion addition. This phenomenon is attributed to enhanced light reflection at oil–water interfaces within soy protein-stabilized emulsions, resulting in a characteristic milky-white appearance (Figure 1). When incorporated into the interstitial spaces of the surimi gel network, these emulsions effectively amplify whiteness [33]. Moreover, the pre-emulsification approach promotes a more homogeneous gel microstructure, optimizing light reflectance through reduced photon scattering losses and thereby improving whiteness [34].

### 3.4. Texture Profile Analysis (TPA) of Surimi Gels

Textural properties serve as critical quality indicators for surimi products, reflecting structural characteristics of the gel network and directly influencing consumer preference and acceptance [35]. As shown in Table 2, increasing emulsion concentrations induced significant reductions (*p* < 0.05) in the hardness, cohesiveness, springiness, and chewiness of surimi gels. At 15% emulsion incorporation, both emulsion systems induced statistically significant reductions in surimi gel hardness compared to the control (E0) (*p* < 0.05). This mechanical weakening is mechanistically attributed to the substantial emulsion droplets diminishing direct protein–protein interactions while simultaneously facilitating interfacial sliding within the myofibrillar protein matrix, collectively promoting structural deformation that manifests macroscopically as reduced gel strength. Notably, 10% EM60 addition significantly enhanced springiness by 0.07 units compared to direct oil phase incorporation (*p* < 0.05), demonstrating the textural improvement from pre-emulsification. However, the two soy protein-stabilized emulsions demonstrated comparable performance in modifying gel texture.

### 3.5. Gel Strength of Surimi Gels

**Figure 6:**
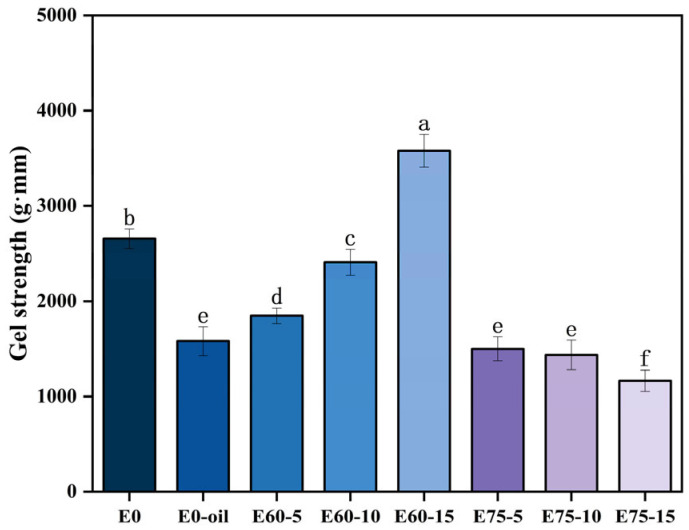
Gel strength variation in surimi gels with different emulsion additives. Different lowercase letters within the same column indicate statistically significant differences (*p* < 0.05).

As shown in the gel strength measurements(Figure 6), surimi gels incorporating EM60 emulsion demonstrated significantly higher gel strength compared to those with direct 60% oil phase addition (E0-oil; *p* < 0.05). Yuan et al. [36] reported similar findings, where direct sturgeon oil incorporation degraded sturgeon surimi gel quality. Direct oil addition likely disrupts protein–protein interactions within the gel matrix, resulting in weakened network structures. Emulsion droplets with smaller dimensions effectively fill matrix voids, forming compact networks through disulfide bonds and hydrophobic interactions with myofibrillar proteins during thermal treatment, thereby enhancing structural integrity [37].

Soy protein isolates in emulsions act as molecular bridges, facilitating oil–myofibril crosslinking through enhanced interfacial interactions. Emulsion incorporation effectively mitigates gel deterioration observed with direct oil addition. Conversely, the gel strength of EM60-modified surimi increased progressively with emulsion concentration, peaking at 15% addition. This enhancement likely stems from smaller EM60 droplets facilitating disulfide bond formation among surimi polypeptides during heating, which promoted myosin aggregation into protein assemblies (gel network) [38]. High internal phase emulsion (HIPE, EM75) concentration showed negative correlation with gel strength, likely due to reduced protein particle density in fixed aqueous phases limiting crosslinking sites and network connectivity. Conversely, larger droplets in high internal phase emulsion (EM75) disrupted disulfide bond formation, inhibiting intermolecular crosslinking of myofibrillar proteins. consequently reducing gel strength and hardness [39]. Nevertheless, HIPEs remain valuable modifiers for developing soft-textured surimi products targeting pediatric and geriatric consumers.

### 3.6. Water-Holding Capacity (WHC) of Surimi Gels

**Figure 7:**
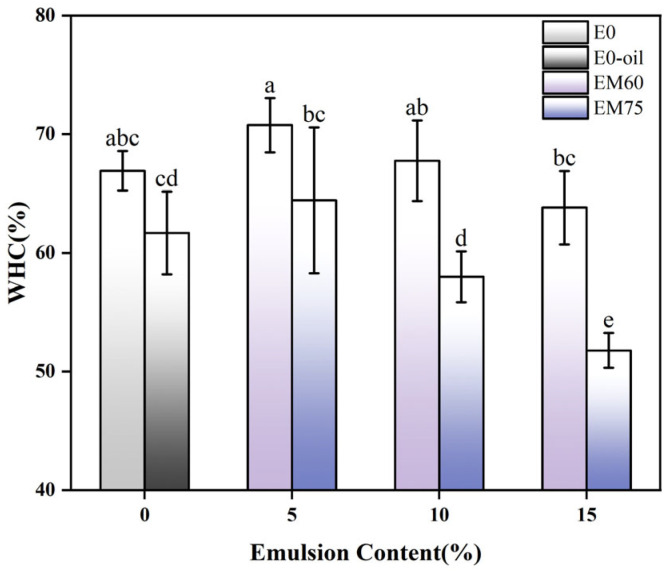
Water-holding capacity of surimi gels modified with various emulsions. Different lowercase letters within the same column indicate statistically significant differences (*p* < 0.05).

Water-holding capacity (WHC) reflects the water-binding ability of protein gel networks in surimi products, with crosslinking degree positively correlating with WHC [40]. Figure 7 reveals that the E0-OIL group exhibited significantly reduced WHC compared to E60-10 (*p* < 0.05), indicating direct oil phase addition compromises gel hydration capacity. However, the interfacial protein layer (soy protein) in emulsions contains hydrophilic groups for enhanced water binding, while mitigating oil–water repulsion to reduce moisture loss [41,42].

However, both surimi gels exhibited a declining trend in water-holding capacity (WHC) with increasing emulsion content. Excessive droplet filling induced droplet aggregation in the emulsions, impairing interactions with hydrophilic groups of soy protein. Notably, a negative correlation was observed between gel strength and WHC in the E60 group. This was attributed to uniform EM60 droplets promoting disulfide bond conversion and enhancing post-heating gel strength. Beyond a critical addition threshold, however, excessive droplets increased network porosity, reducing WHC. In contrast, the E60 group showed significantly greater WHC enhancement than E75 (*p* < 0.05). This phenomenon arose from larger droplet sizes in high internal phase emulsion (EM75), which disrupted the gel network. Conversely, smaller EM60 droplets strengthened hydrophobic interactions with myosin during heating, improving water retention. Increased gel strength further corroborated these mechanisms. Thus, soy protein-based emulsions with 60% oil phase as fat substitutes demonstrated superior WHC and gel strength in surimi gels.

### 3.7. Low-Field Nuclear Magnetic Resonance (LF-NMR) and Magnetic Resonance Imaging (MRI) of Surimi Gels

**Figure 8:**
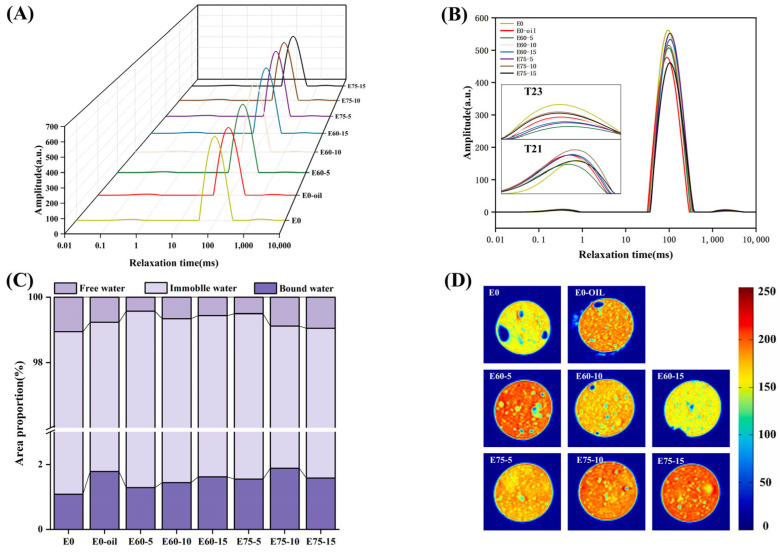
Moisture distribution characteristics in emulsion-modified surimi gels: (**A**) 3D relaxation time curves; (**B**) 2D relaxation time curves; (**C**) area proportions of relaxation peaks; (**D**) hydrogen proton pseudocolor mapping.

LF-NMR characterizes water state transitions in surimi gels by analyzing hydrogen nucleus relaxation properties under magnetic fields. Figure 8 displays T_2_ relaxation time distributions across experimental groups. Peak T_21_ corresponds to bound water, T_22_ to immobile water (strongly correlated with WHC), and T_23_ to free water residing outside the myofibrillar crystalline lattice [43]. As shown in Figure 8A,B, emulsion-modified groups exhibited significantly reduced T_23_ peak areas compared to controls, indicating decreased free water content. The E60-10 group demonstrated 23.7% lower T_23_ area ratios than E0-oil counterparts. This aligns with findings by Zhang et al. [44], where emulsion incorporation yielded lower free water levels than direct soybean oil addition.

At 5% emulsion concentration (Figure 8C), E60 gels showed minimal T_23_ areas, suggesting enhanced water phase conversion from free to bound/immobile states. Figure 8C illustrates emulsion-induced water redistribution, with substantial free-to-bound/immobile water transitions. The superior T_23_ reduction in E60 versus E75 systems (*p* < 0.05) stems from enhanced gel matrix–water interactions mediated by 60% oil phase soy protein emulsions, facilitating macromolecular water binding [45]. These T_23_ findings correlate strongly with WHC measurements, confirming methodological consistency.

MRI proton density maps (Figure 8D) visually resolve moisture distribution patterns within surimi matrices. Emulsion-modified gels demonstrated elevated moisture content with more homogeneous distribution (reduced blue speckling) versus controls. In E60-modified gels, proton intensity decreased with increasing emulsion concentration, correlating with moisture loss and distribution heterogeneity. E60-10 showed constrained red signal distribution compared to E0-OIL, potentially reflecting soybean oil’s proton signal amplification in direct addition systems [45]. Conversely, E75 systems exhibited paradoxical proton signal intensification with emulsion loading, suggesting moisture retention at the expense of distribution uniformity.

### 3.8. Rheological Properties of Surimi Sols

#### 3.8.1. Shear Rate Sweep Analysis

**Figure 9:**
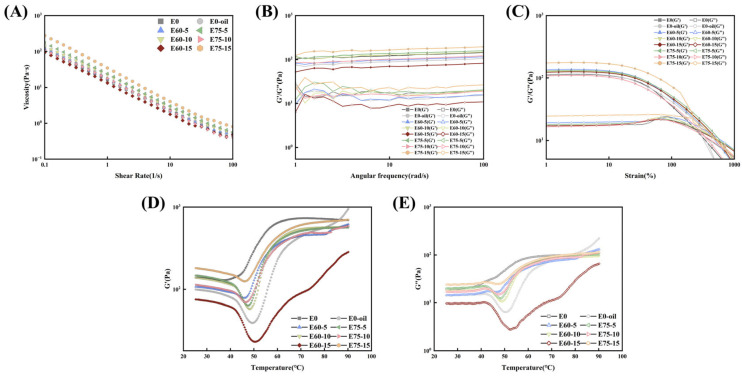
Rheological behavior of surimi sols with emulsion additives: (**A**) shear viscosity sweep; (**B**) frequency sweep; (**C**) strain sweep; (**D**,**E**) temperature sweep.

Shear rate sweeps (Figure 9A) revealed pronounced shear-thinning behavior across all samples, with apparent viscosity (Pa·s) decreasing exponentially as shear rate increased. The 15% HIPE-modified sol exhibited maximum viscosity retention, likely attributable to the superior thickening capacity of high internal phase emulsions (HIPEs) at elevated concentrations, confirming their stabilizing efficacy. The Ostwald–de Waele model fitting of viscosity-shear rate data yielded power-law parameters summarized in Table 3. Correlation coefficients (R^2^ > 0.99) confirmed excellent model–data agreement. E75 systems demonstrated higher consistency coefficients (K) than E60 at 5% (+7.258) and 15% (+22.350) concentrations, while E60 surpassed E75 by 4.443 at 10% loading. Both rheograms and power-law parameters corroborated that pre-emulsified systems outperformed direct oil phase addition in viscosity and K-value retention. These findings position soy protein HIPEs as effective fat substitutes for silver carp surimi sols, providing enhanced viscosity modulation. These findings position soy protein HIPEs as effective fat substitutes for silver carp surimi sols, providing enhanced viscosity modulation.

#### 3.8.2. Strain Sweep and Frequency Sweep Analysis

Strain sweep analysis identified the linear viscoelastic region (LVR). As shown in Figure 9C, all surimi sol samples maintained relatively stable G′ and G” values within 1–10% strain range. Beyond this threshold, all formulations exhibited yielding behavior characterized by G′ reduction and G″ elevation post 10% strain. Emulsion-modified sols demonstrated delayed strain crossover points under high deformation, reflecting substantial microstructural differences induced by soy protein-stabilized emulsions [46].

Frequency sweep results (Figure 9B) revealed ascending G′ dominance over G” across 1–100 rad/s, likely attributable to interfacial reorganization of emulsion particles enhancing elastic responses at elevated frequencies. Emulsified systems exhibited higher G′ than direct oil-incorporated counterparts. While E75 (5–15% HIPE addition) showed superior G′ to E60, this trend reversed at 10% loading where E60 demonstrated enhanced elasticity. This phenomenon may arise from critical-phase interactions between soy-stabilized HIPE and surimi proteins at specific concentrations, temporarily impeding network crosslinking. Similar transitional behavior was documented in Yu et al.’s investigation [47]. Phase inversion (dispersed-to-continuous transition) occurred at 15% loading, establishing an emulsion-particle-reinforced composite network. The synergistic “self-supporting effect” of particles and protein matrix consequently restored structural rigidity [48].

Dynamic rheological parameters were determined through power-law model fitting (Table 4). Correlation coefficients (R^2^) exceeding 0.90 confirmed satisfactory model–data alignment. Positive b values verified favorable viscoelastic properties across all samples. The a parameter (indicating network strength through physical protein interactions) followed identical trends to storage modulus observations. Table 5 further evaluates structural rigidity (t) and network extensibility (z), showing consistency with power-law derived parameters. The 1.241-unit z-value enhancement in E60-10 versus E0-OIL reaffirms emulsion pre-incorporation efficacy in optimizing gel network distribution.

#### 3.8.3. Temperature-Dependence of Gel Rheology

Temperature sweep analysis effectively monitors dynamic viscoelastic changes during surimi’s sol–gel transition. Figure 9D,E demonstrate predominant G′ over G″ values throughout the heating protocol (25–90 °C) across all samples. The heating profile typically exhibited three characteristic stages (setting, modori, and kamaboko) as defined by G′ evolution [49]. Stage I (25–40 °C) showed gradual G′ decline, potentially resulting from myofibrillar protein dissociation and subsequent peptide chain realignment. Stage II witnessed accelerated G′ reduction, reaching minimum values at 55 °C with progressive heating. This phenomenon likely stems from myosin tail extension limiting protein aggregation, coupled with endogenous protease activation at critical temperatures that promote proteolytic degradation [50,51]. Subsequent heating induced thermal-driven protein crosslinking, manifested by progressive G′ increase and eventual formation of irreversible 3D gel networks.

### 3.9. Microstructure of Surimi Gels

**Figure 10:**
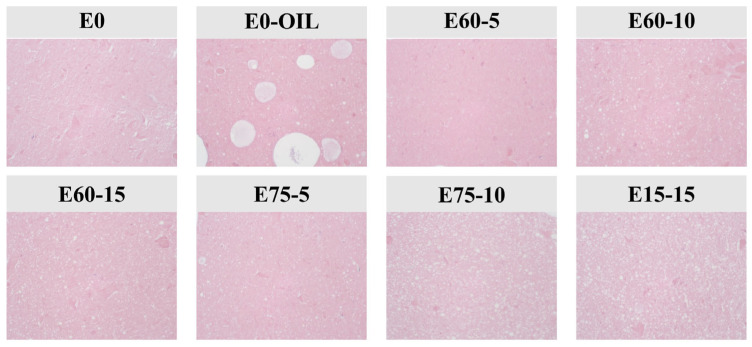
Crosslinking architectures of surimi gels under different additive conditions.

Optical microscopy revealed the distribution of emulsion droplets within myofibrillar protein networks, demonstrating the microstructure of surimi gels. As shown in Figure 10, emulsion-containing gels exhibited more uniform structure compared to non-emulsion controls. E0-oil gels displayed irregular large oil droplets that increased interfibrillar spacing, creating enlarged pores and disrupting the gel network. This phenomenon may result from inadequate emulsification between oil phase and soy protein particles, leading to oil droplet aggregation during thermal gelation that interferes with myofibrillar proteins [52].

Under identical parameters (oil content and emulsion dosage), E60-10 formed uniformly distributed small droplets that established dense 3D networks through enhanced protein crosslinking, thereby improving gel strength and water-holding capacity. High internal phase emulsion (EM75)-modified gels exhibited more compact and irregular droplet arrangements compared to EM60-modified gels. Increasing EM75 concentration caused droplet crowding in the gel matrix, which inhibited protein crosslinking. These observations corroborated the gel strength and water-holding capacity (WHC) results, indicating that droplet size, emulsion dosage, and oil phase content collectively regulate gel properties through their spatial interactions within protein networks.

## 4. Conclusions

The addition method of pre-emulsifying soybean oil into emulsion enhanced the structural rigidity of surimi sol while retaining the lipid flavor inside the surimi gel, improved the water-holding capacity, gel strength, hardness, and free water-binding ability of silver carp surimi gel, made the three-dimensional network structure more compact. In summary, soy protein-stabilized emulsions with 60% and 75% oil phases exhibit distinct functionalities in surimi gel systems. EM60 enhanced gel strength and water retention, while EM75 improved whiteness and extensibility. These findings support their use as healthier fat substitutes. Future studies should explore sensory acceptance, shelf-life stability, and industrial scalability.

## Figures and Tables

**Table 1:** Summary of protein and oil contents in the emulsions.

Groups	SPI Content (%)	Soy Oil Content (%)
EM10	90	10
EM20	80	20
EM40	60	40
EM60	40	60
EM75	25	75

**Table 2:** Texture profile analysis (TPA) characteristics of emulsion-modified surimi gels.

Sample	Hardness (g)	Springiness	Cohesiveness	Chewiness (g·mm)
E0	3973.72 ± 334.22 ^a^	0.9 ± 0.02 ^ab^	0.81 ± 0.02 ^a^	2888.35 ± 226.75 ^a^
E0-oil	3404.55 ± 762.63 ^abcd^	0.84 ± 0.13 ^b^	0.79 ± 0.03 ^ab^	2300.2 ± 702.37 ^b^
E60-5	3905.32 ± 388.65 ^ab^	0.9 ± 0.02 ^ab^	0.81 ± 0.01 ^a^	2853.71 ± 286.12 ^a^
E60-10	3303.08 ± 209 ^bcd^	0.91 ± 0.01 ^a^	0.82 ± 0.01 ^a^	2445.13 ± 163.74 ^ab^
E60-15	3009.19 ± 597.02 ^d^	0.93 ± 0.04 ^a^	0.81 ± 0.01 ^a^	2252.94 ± 457.45 ^b^
E75-5	3963.22 ± 199.25 ^a^	0.91 ± 0.02 ^a^	0.8 ± 0.01 ^ab^	2537.66 ± 173.63 ^ab^
E75-10	3678.15 ± 493.87 ^abc^	0.87 ± 0.05 ^ab^	0.78 ± 0.02 ^b^	2497.14 ± 387.24 ^ab^
E75-15	3096.79 ± 402 ^cd^	0.89 ± 0.03 ^ab^	0.79 ± 0.03 ^b^	2166 ± 292.01 ^b^

Note: Different lowercase letters within the same column indicate significant differences in TPA properties (*p* < 0.05).

**Table 3:** Fitted power-law parameters k and n for different surimi gel samples.

	$\eta =k{\left(\gamma \right)}^{n-1}$		
Sample	K	n	R^2^
E0	15.555	0.139	0.99985
E0-OIL	18.119	0.154	0.99953
E60-5	16.968	0.142	0.99983
E60-10	21.913	0.197	0.99977
E60-15	13.290	0.151	0.99983
E75-5	24.226	0.143	0.99986
E75-10	17.470	0.209	0.99964
E75-15	35.640	0.106	0.99983

**Table 4:** Fitted power-law parameters a and b for different surimi gel samples.

	$G^{\prime }=a{\left(\omega \right)}^{b}$		
Sample	a	b	R^2^
E0	104.406	0.069	0.98156
E0-OIL	73.505	0.079	0.97003
E60-5	81.23	0.077	0.97327
E60-10	106.324	0.071	0.94841
E60-15	58.108	0.075	0.92261
E75-5	113.304	0.075	0.94144
E75-10	83.695	0.082	0.98609
E75-15	141.552	0.069	0.9039

**Table 5:** Fitted power-law parameters t and z for different surimi gel samples.

	$G^{*}=t{\left(\omega \right)}^{\frac{1}{z}}$		
Sample	t	z	R^2^
E0	105.928	14.808	0.97691
E0-OIL	74.972	13.047	0.9736
E60-5	82.668	13.382	0.97647
E60-10	107.714	14.288	0.94019
E60-15	59.138	13.912	0.91052
E75-5	115.462	13.978	0.94828
E75-10	85.192	12.541	0.98257
E75-15	143.992	14.972	0.8936

## Data Availability

The data presented in this study are available on request from the corresponding author (due to privacy or ethical restrictions).
